# Preparation and characterization of acrylic resins with bioactive glasses

**DOI:** 10.1038/s41598-022-20840-1

**Published:** 2022-10-05

**Authors:** Zbigniew Raszewski, Katarzyna Chojnacka, Marcin Mikulewicz

**Affiliations:** 1 SpofaDental, Markova 238, 506-01 Jicin, Czech Republic; 2 Department of Advanced Material Technologies, Wroclaw University of Science and Technology, Wrocław, Poland; 3 Division of Facial Abnormalities, Department of Dentofacial Orthopedics and Orthodontics, Medical University of Wroclaw, Wrocław, Poland

**Keywords:** Biological techniques, Chemical biology, Medical research, Chemistry

## Abstract

This study aimed to prepare a bioactive acrylic material by adding different types of glasses. Commercially available polymerized acrylic resin was mixed with 10% of four different types of glasses in the powder form and cured. Flexural strength, sorption, and solubility of the samples were tested according to ISO 20795-1:2013. The total number of samples used in the tests were 60. The materials were placed in artificial saliva of pH 4 and 7, and elution was performed for 0, 1, 28, and 42 days. The collected samples were analyzed using inductively coupled plasma atomic emission spectrometry to detect Ca, P, and Si ions and using ion chromatography to detect F ions. The materials obtained after modification with glasses showed lower compressive strength compared with pure polymethyl methacrylate but met the standard requirements. Two glass types showed higher solubility values compared with the value defined by the ISO standard. Biomin C and S53P4 released Ca, P, and Si ions, respectively, after 42 days in artificial saliva. Acrylic resins modified with 10% Biomin C and S53P4 glasses can be a valuable source of Ca and P ions under acid conditions for 28 and 42 days.

## Introduction

Despite the emergence of new alternatives, acrylic materials are still most widely used for the fabrication of removable dentures in prosthetic dentistry. This popularity is due, among others, to their long-term use and ease of processing in dental technology laboratories^1,2^. Acrylic materials are characterized by good optical properties and biocompatibility^3^. Unfortunately, besides several advantages, acrylic resins possess some disadvantages, such as the restricted saliva flow within the denture base area. Wearing dentures, eating food, and thus reduced saliva flow in the area of the remaining teeth, causes a decrease in pH, which in turn is a factor that may cause tooth changes in the remaining teeth. Saliva flow is disturbed at the locations where the acrylic material contacts soft tissues or teeth^4,5^. Therefore, acrylic resins should be modified to increase their bioactivity .

In the scientific literature, there are extensive discussions on the meaning of the term “bioactive material,” but according to the IUPAC (International Union of Pure and Applied Chemistry) recommendations from 2012, it is defined as a “material which has been designed to induce specific biological activity;” in other words, a material that evokes a response from a living organism can be called a bioactive material^6^.

One of the existing strategies to modify acrylic materials and form bioactive materials is the addition of various types of nanomaterials such as silver and titanium oxide^7–10^. Another strategy is the addition of various types of medicinal substances, such as antibiotics^11^ and chlorhexidine^12,13^. In addition to these approaches, the use of different types of bioactive glasses can also enable the modification of acrylic materials. This type of ceramics undergoes gradual hydrolysis under the influence of water and releases various types of ions into the environment, such as fluorine and phosphate anions or calcium cations^6^. This strategy is already widely applied in glass ionomer cement, as well as in composite filling^14–17^ and orthodontic adhesives^18^. Alkaline cations increase the pH, and fluorine anions have proven cariostatic effects. Eluted ions, as in the case of glass ionomer cement, can perform the remineralization function^17–19^. However, they can completely hydrolyze at a lower pH and thus cannot form hydroxyapatite (HA); the minimum pH value for the formation of HA is 4.5–5.5 depending on the individual^20^.

So far, these glasses have been successfully used in glass ionomer cement, composites, and toothpaste (Biomin)^18^. For example, Bioglass 45S5 and S53P4 were first synthesized in the late 1970s and have been in clinical use since 1985^21^. The addition of CaF_2_ allows glass to release fluorine ions. However, excessive addition of CaF_2_ leads to uncontrolled crystallization of crystalline phases, including the formation of cuspidine, and fluoride ions. Therefore, Biomin C glass, which contains chlorine ions, was developed in 2015^20^. From a dental perspective, chlorapatite will be completely converted to HA in the presence of water^22^. Biomin F is an example of fluorine glass used in these tests.

This study aimed to synthesize an acrylic material that exhibits bioactive properties by releasing calcium, phosphorus, and fluorine ions.

Based on similar systems of methacrylate resins present in composite materials, it was hypothesized that the addition of special types of glasses to poly (methyl methacrylate) (PMMA) can result in a material with bioactive properties.

## Materials and methods

Bioactive glass samples were prepared by Cera Dynamic (Kent, England) by melting certain oxides at a temperature of 1500 °C for 1 h and then rapidly cooling them in distilled water (melt quench route)^21^. Then, the samples were ground to obtain particles of size d_50_ = 5 μm. These powder samples were used in the present study.

Acrylic resin samples were prepared by mixing 10% bioactive glasses (by mass, Table 1) with PMMA resins (Superacryl Plus, SpofaDental, Czech Republic). To achieve a homogenous mixture, a ball mill (Jezirska Porcelana, Czech Republic) was used, with a rotation speed of 40 rpm. A homogenous mixture was obtained with 10 g of glass and 90 g of PMMA by mixing them using ceramic balls (300 g) for 2 h.

**Table 1:** Composition of glass used in this study to prepare a 10% mixture, according to the information provided by the supplier.

	SiO_2_	P_2_O_5_	CaO	Na_2_O	CaF_2_	CaCl_2_
S53P4	53.8%	1.7%	21.8%	22.7%	0	0
Biomin F	36–40%	4–6%	28–30%	22–24%	1.5–3.0%	0
45S5	46.1%	2.6%	26.9%	24.4%	0	0
Biomin C	30.3–31.8%	5.0–5.3%	44.1–46.3%	0	0	16.7–20.6%

The materials (PMMA and bioactive glasses) were then mixed with methyl methacrylate and subjected to thermal polymerization. Superacryl Plus resin (SpofaDental, pure PMMA) was used as the reference material. In all the samples tested, the powder-to-liquid ratio was 2/1 [g], and thermal polymerization was performed according to the instructions of the manufacturer (Superacryl Plus, SpofaDental) according to the well-known, flasking technique. Using the lost wax method, six molds for acrylic plastic were produced inside the plaster of Mramorit Blue (SpofaDental, Czech Rep). All the molds were placed on a laboratory press, and the excess material was removed by applying a pressure of 2000 kg/mm^2^ for 10 min.

**Figure 1:**
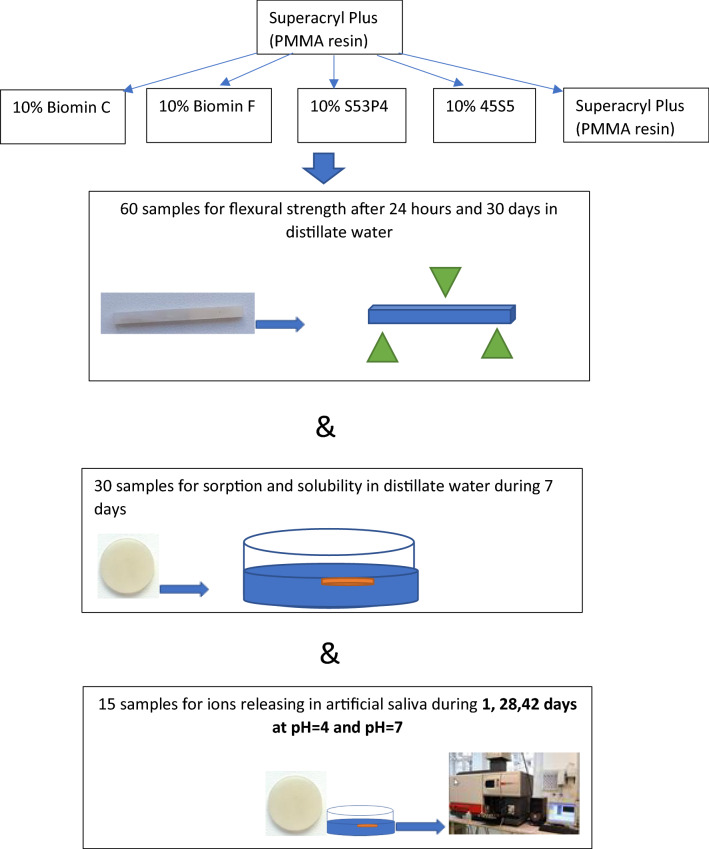
Scheme of sample preparation and testing.

The samples were kept in water at a frame at 60 °C, and the temperature was increased to 100 °C in 30 min. Finally, the polymerization material was kept at 100 °C for 1 h and then gradually cooled. After polymerization, the samples were removed from the molds, polished with sandpaper (200, Kolo, Poland), and used for further testing. The schematic of the procedure is presented in Fig. 1.

### Flexural strength

Sorption and solubility were analyzed according to ISO 20795-1: 2013 (en), Dentistry—Denture base polymers^23^. In total, 60 pieces were evaluated for flexural strength, whose dimensions were 65 × 10 × 3.3 mm. These samples were then stored in distilled water for 24 h and 30 days. The medium was changed every 3 days.

Mechanical resistance to fracture was determined using three-point deflections (50-mm supports), and the breaking head was set at 5 mm/min. Sixty samples were prepared for the tests (six for each type of material). Pure PMMA was used as the reference material.

### Sorption and solubility

According to ISO 20795-1: 2013 (en), Dentistry—Denture base polymers, sorption and solubility in distilled water in the first 7 days are important parameters that determine the standard of acrylic resins^23^. Thirty samples with a diameter of 50 mm and a thickness of 1 mm prepared from acrylic resin and 10% bioactive glasses were subjected to this test. Disks made of PMMA were used as the reference material.

Sorption (*A*) and solubility (*B*) were calculated based on Eqs. (1) and (2):

1 $$A=\frac{M2-M1}{S}$$

2 $$B=\frac{M1-M3}{S}$$

where M2 is the mass of the sample after 7 days of immersion in distilled water, M1 is the mass before immersion in water, M3 is the mass of the material after immersion in water and drying in the exicator, and S is the volume of the disk measured using a calibrated caliper.

### Ion release

To analyze the bioactive properties of the samples, ions were released from acrylic materials in artificial saliva at pH 4 and 7. Disks with a diameter of 5 mm and a thickness of 1 mm were prepared for the tests and were thermally polymerized as described earlier. The total number of samples in this study was 15 (three for each material type). Disks made of PMMA were used as the reference material.

### Preparation of the artificial saliva solution

The artificial saliva solution was prepared by dissolving sodium chloride (0.4 g) (NaCl, Sigma Aldrich, Poland), potassium chloride (1.21 g) (KCl, Sigma Aldrich, Poland), hydrated potassium dihydrogen phosphate (0.78 g) (NaH_2_PO_4_ × 2H_2_O, Sigma Aldrich, Poland), hydrated sodium sulfide (0.12 g) (Na_2_S × 9H_2_O, Sigma Aldrich, Poland), and urea (1.0 g) (Sigma Aldrich, Poland) in ultrapure water (1000.0 g) (Merck, Germany). The prepared solution was transferred to two vessels and adjusted to pH 4 and 7 using hydrochloric acid (0.1 Mol) and sodium hydroxide (0.1 Mol), respectively (both from Merck, Germany)^24^.

### Extraction

The samples obtained from the dental materials (three samples of each glass and acrylic resins as reference, diameter 5 mm, thickness 1 mm) were extracted in artificial saliva at pH 4 and 7. A disk of each material was kept in polypropylene vessels and covered with 5 mL of artificial saliva solution. After extraction, a sample of the material was transferred to a new container and replenished with new artificial saliva. The prepared samples were placed on a shaker and incubated at 37 °C (Sigma Aldrich, Poland). The number of the prepared replicates was adjusted to end the extraction process after 1, 28, and 42 days, respectively. After the extraction process, the samples were acidified using trace pure nitric acid (Merck, Germany) and made up to a volume of 20 mL. The blank and extracted samples were then used in the multielement analysis.

### Mineralization of the samples

The acrylic resins (five samples) were decomposed using the two-stage wet mineralization method in a closed system assisted by microwaves, using the START D microwave decomposition system (Milestone, Italy). The comminuted samples, each weighing approximately 0.1 g, were placed in a Teflon vessel, and the process was carried out in two stages. In the first stage, 3 mL each of ultrapure demineralized water and sulfuric acid (Merck, Germany) was added to each sample. Mineralization was carried out for 10 min at 100 °C with 1000 W oven power. In the second step, trace pure nitric acid (5.0 mL) (Merck, Germany) was added to the Teflon vessels with permineralized materials. The process was carried out for 35 min at 200 °C with 1000 W oven power. After the process ended, the cooled minerals were transferred to bottles made of an HDPE (high density polyethylene) material and diluted to a weight of approximately 50 g.

### Elemental analysis

The elemental composition of saliva extracts and pure acrylic resins was analyzed following the inductively coupled plasma atomic emission spectrometry method using the iCAP 6500 Duo optical spectrometer with horizontal and vertical plasma (Thermo Fisher Scientific, USA). The spectrometer was equipped with an ultrasonic U5000AT + nebulizer (CETAC, USA), which showed about tenfold lower detection limits of the concentration of the samples. The extracts and pure samples of acrylic resins were analyzed using the validated research methods in Chemical Laboratory of Multielement Analyses, accredited by the Polish Center for Accreditation (AB696).

### Fluoride content analysis

The concentration of fluoride ions in the extracts was determined by ion chromatography using a Dionex ICS 1100 ion chromatograph (Thermo Fisher Scientific, USA). The dental material extract was injected directly through a sterile 0.2-µm syringe filter into the chromatography column. The concentration of fluoride ions was measured with a retention time of approximately 2.5 min.

Statistical analysis was performed by one-way ANOVA using the Tukey HSD Test Calculator available at Astasta.com. For all tests, the confidence level was assumed at *p* < 0.05.

## Results

Flexural strength is one of the most important mechanical parameters for acrylic resins used in the production of denture bases, which determines whether a denture made of such a material will not crack during use. Table 2 shows the flexural strength of acrylic samples modified with four types of bioactive glasses after 24 h and 30 days.

**Table 2:** Flexural strength [MPa] after 24 h and 30 days for four different types of glasses and PMMA resin (Superacryl Plus) as the reference.

	45S5	Biomin F	S53P4	Biomin C	Superacryl Plus
**24 h**					
AVG	76.39	78.05	77.96	76.01	83.13
SD	± 3.63	± 5.91	± 2.29	± 3.37	± 2.85
	*p* < 0.01			*p* < 0.01	
**30 days**					
AVG	68.82	70.31	70.23	68.48	74.89
SD	± 3.27	± 5.32	± 2.06	± 3.04	± 2.57
	*p* < 0.05			*p* < 0.05	

Flexural strength for samples 45S5 and Biomin C is statistically significant at the confidence level of *p* < 0.01 compared with the reference Superacryl Plus.

After 30 days of immersion, the same flexural strength for samples 45S5 and Biomin C is statistically significant at the confidence level of *p* < 0.05 compared with the reference Superacryl Plus.

The results showed only a 10% reduction in the flexural strength of all samples with bioactive glasses. The highest flexural strength was observed for samples with glass S53P4 and Biomin C.

After 30 days, a further reduction in the flexural strength was observed compared with the results obtained after 24 h. All results were higher than 65 MPa, which is the minimum requirement for denture base materials according to the ISO standard.

The materials used in the preparation of the denture plate should have low sorption and solubility. These two factors are responsible for the absorption of food debris and saliva and its discoloration. In the case of bioactive materials, solubility may be one of the determinants of the release of ions into the oral environment. The values of sorption and solubility observed after 7 days in distilled water are presented in Table 3.

**Table 3:** Sorption and solubility [μg/mm^3^] tested in distilled water for 7 days for four bioactive glasses and Superacryl Plus as the reference material.

Material	Solubility [µg/mm]	sorption [µg/mm^3^]
Biomin f	3.29 ± 0.52 *p* < 0.01	9.48 ± 0.81
Biomin c	1.43 ± 0.37	12.38 ± 0.92
s53p4	1.53 ± 0.29 *p* < 0.01	15.57 ± 1.56 *p* < 0.01
45s5	4.92 ± 0.46 *p* < 0.01	19.19 ± 1.76 *p* < 0.01
Superacryl plus	0.50 ± 0.12	10.16 ± 1.40

The solubility of all samples modified with bioactive glasses in relation to Superacryl Plus is statistically significant at the confidence level of *p* < 0.01.

The sorption of acrylic samples modified with bioactive glasses in relation to the reference sample has a confidence level of *p* < 0.01 for 45S5 and S53P.

The highest solubility was observed for Biomin F and 45S5: 3.29 ± 0.52 and 4.94 ± 0.46 μg/mm^3^, respectively. These values indicate that these materials can be soluble in water and are potential sources of ions.

The values of leaching of calcium, phosphate, and fluoride ions are presented in Table 4.

**Table 4:** Results of ions released after a long period in artificial saliva. The phosphate and silica ion values were calculated from the difference between the test result and the blank values for the respective pH.

	Ca [mg/L]	P [mg/L]	Si [mg/L]	F [mg/L]
**BLANK**				
pH 4	0	20.46 ± 5.32	0.01 ± 0.0	0
pH 7	0	20.48 ± 5.75	0.27 ± 0.04	0
**BIOMIN F**				
pH 4 1 day	3.33 ± 0.5	35.50 ± 5.33	2.52 ± 0.38	7.05 ± 1.06
28 days	0	30.66 ± 4.60	10.46 ± 1.57	0
42 days	0	31 ± 4.6819	11.7 ± 1.76	0.18 ± 0.03
pH 7 1 day	1.28 ± 0.19	35.22 ± 5.28	2.37 ± 0.36	0.30 ± 0.05
28 days	0	31.43 ± 4.71	9.01 ± 1.35	0.32 ± 0.05
42 days	0	16.60 ± 2.49	9.07 ± 1.36	0.77 ± 0.12
PMMA*				
pH 4 1 day	0	20.45 ± 5.00	0.03 ± 0.00	0
28 days	0.03 ± 0.05	20.49 ± 4.90	0.12 ± 0.02	0
42 days	0.05 ± 0.01	20.44 ± 4.79	0.12 ± 0.02	0.00
pH 7 1 day	0.2 ± 0.01	20.42 ± 4.44	0.26 ± 0.04	0
28 days	0.02 ± 0.01	20.49 ± 4.75	0.35 ± 0.05	0
42 days	0	20.44 ± 4.87	0.45 ± 0.07	0
**S53P4**				
pH 4 1 day	1.92 ± 0.29	30.91 ± 4.64	0.94 ± 0.14	0
28 days	1.40 ± 0.21	28.93 ± 4.34	5.21 ± 0.78	0
42 days	6.17 ± 0.92	28.57 ± 4.29	10.85 ± 1.63	0
pH 7 1 day	1.48 ± 0.22	28.56 ± 4.26	1.71 ± 0.26	0
28 days	1.54 ± 0.08	27.33 ± 4.10	5.40 ± 0.81	0
42 days	3.77 ± 0.57	29.73 ± 4.46	8.89 ± 1.33	0
**45S5**				
pH 4 1 day	2.71 ± 0.41	29.88 ± 4.48	1.92 ± 0.29	0
28 days	1.38 ± 0.06	26.02 ± 3.9	9.94 ± 1.49	0
42 days	1.04 ± 0.16	26.21 ± 3.93	9.64 ± 1.45	0
pH 7 1 day	1.73 ± 0.26	26.75 ± 4.01	2.72 ± 0.41	0.00
28 days	0.62 ± 0.09	23.52 ± 3.53	7.08 ± 1.06	0.00
42 days	0.06 ± 0.01	14.91 ± 2.24	6.2 ± 0.93	0.00
**BIOMIN C**				
pH 4 1 day	6.50 ± 0.97	27.47 ± 4.12	1.00 ± 0.15	0.00
28 days	11.96 ± 1.79	25.88 ± 3.88	4.43 ± 0.66	0.00
42 days	2.43 ± 0.36	24.05 ± 3.61	3.02 ± 0.21	0.00
pH 7 1 day	4.90 ± 0.74	27.45 ± 4.12	1.58 ± 0.24	0.00
28 days	5.04 ± 0.76	26.71 ± 4.01	2.09 ± 0.31	0.00
42 days	1.19 ± 0.18	23.76 ± 3.56	4.36 ± 0.65	0.00

*Phosphate and a small amount of silicon and calcium come from saliva as well as from the surface of the samples, which were thermally polymerized in tap water.

The results obtained indicate that the acrylic system, together with the bioactive glass added, can release ions into the reaction environment and thus act as a source of raw materials for the production of HA.

The ion release rate depends on the type of glass used. In the case of Biomin F, virtually all the available calcium cations were washed out of the samples within the first 24 h. However, Biomin C can be a valuable source of calcium cations (11.96 ± 1.79 mg/L) and phosphate anions (25.88 ± 3.88 mg/L) under acid conditions (pH 4). For S53P4 glass, the highest ion release values were observed after 42 days at pH 4.

In Biomin F, fluoride ions were readily available and leached rapidly within the first 24 h at pH 4. On the other hand, in a neutral environment (pH 7), fluoride ions were released in smaller amounts, but over a longer period of time.

## Discussion

The history of the synthesis of bioactive glasses dates long back, and attempts to produce bioactive glasses were started in 1970 when Hench used a small amount of CaF_2_ as a raw material in glass composition instead of a certain amount of CaO and Na_2_O during glass melting. However, he discovered that the ability to form apatite decreased with an increase in the calcium fluoride concentration. This kindled the interest in the production of glasses with bioactive properties, which are now widely used in cement, glass ionomer, and composites^22^.

Composite materials are closely related to acrylic resins, which contain the simplest high-viscosity methacrylic resin—methyl methacrylate—which, after polymerization, forms a network of PMMA. Therefore, this study aimed to investigate how PMMA-based materials will release ions from bioactive glasses^14^.

The study hypothesis regarding the mechanical properties and ion release of PMMA-based materials with an admixture of bioactive glasses was validated.

Acrylic resins showed a lower flexural strength after the addition of four types of bioactive glasses compared with the unmodified resin. The flexural strength of the sample containing Biomin F (78.05 ± 5.91 MPa) was similar to that of the sample containing pure PMMA (83.13 ± 2.85 MPa). Both these materials met the requirements of the ISO standard for denture base materials. This is because the filler has no chemical connection to the PMMA polymer. A similar scenario was observed using composite materials with nonsilanized fillers^14^. In the study of Bettencourt et al., the addition of another nonbonded substance, chlorhexidine, to Probase Cold acrylic reduced its fracture resistance. However, the addition of chlorhexidine did not reduce the fracture resistance of Kooliner or Ufi Gel Hard materials. Superacryl Plus is a PMMA-based material similar to Probase Cold; therefore, the results of this study are in line with those of Bettencourt et al.^12,13^.

The materials used in dentures are in constant contact with water throughout their use (saliva, and drinks and food consumed). Long-term storage of acrylic materials in water reduces their resistance to breakage. This is attributable to the plasticizing effect of water absorption, which was confirmed in this investigation^25,26^.

As reported in a previous study, the sorption of heat-curing acrylic materials in distilled water or artificial saline ranges from 17.5 ± 0.88 to 27.25 ± 1.04 μg/mm^3^^27^. Our reference material Superacryl Plus showed a sorption value of 10 μg/mm^3^, which may vary with the time duration tested and the polymerization method^28^. As mentioned earlier, the Biomin F sample showed a sorption value of 9.48 ± 0.81 μg/mm^3^. This may prove that Biomin F prevents excessive absorption of water into the material, which is the most desirable characteristic of denture materials.

For any acrylic material, its chemical structure, e.g., the content of various ions, has a great influence on its sorption and solubility. Solubility of an acrylic material can be increased by adding different oxides (ZrO_2_, TiO_2_) that are not chemically bound to PMMA^29–31^.

The high content of sodium ions in acrylic resins modified with glasses may be responsible for their higher solubility and sorption. These ions are quickly washed out and exchanged for H_3_O^+^ ions when the samples are in contact with distilled water^22^.

Similar observation was reported in the study of Khvostenko^19^, in which a composite with bioactive glasses was kept in distilled water for 30 days using a glass with a low Na_2_O content to minimize water uptake, swelling, and possible cracking, which resulted in a significant but reduced flexural strength.

Indeed, the addition of nonpolymer network bonding ions weakens the mechanical properties of the material, but in the case of bioactive materials, there is a compromise between mechanical properties and ion release. The tests carried out by Raszewski^7^ on an acrylic material modified with Fritex and Kavitan showed that the addition of glasses used in glass ionomer cement resulted in the release of fluorine ions for 30 days.

Al-Eesaa^14,18^ and Liu et al.^10^ showed that glass materials can deliver ions from composite materials to nearby tissues; for example, glass 45S5 can form a new layer of HA on the surface of the composite material. The same glass can also release ions from PMMA-based materials as shown in this study.

At a lower pH (pH 4), glass degradation is much more rapid compared with neutral pH. Many authors attribute this phenomenon to the first step of hydrolysis when H^+^ ions from the immersion media were exchanged with Ca^2+^, Sr^2+^, and Na^+^ ions from the glass structure, which is associated with an increase in pH^10,15^.

In the literature, information on two types of bioactive glasses that contain phosphate ions can be found. Edén et al.^30^ and O’Donnell et al.^31^ observed a relationship between the amount and rate of apatite formed under the surface of the composite.

In the present study, the release of ions was faster at acidic pH. However, more silicate and phosphate anions were released at pH 7, which was due to the formation of the corresponding salts.

For example, the samples containing S53P4 glass released 2.43 ± 0.36, 24.05 ± 3.61, and 3.02 ± 0.21 mg/L of Ca^2+^, PO_4_^3−^, and SiO_4_^2−^, respectively, after 42 days at pH 4.

During the second step of glass degradation in the water–saliva solution, the removal of protons from the solution leads to the accumulation of hydroxyl groups, resulting in the release of Ca^2+^ ions into the solution with a simultaneous increase in pH^22^. This was also noticed in the present study after the increase in the amount of calcium ions over a longer period of time for S53P4 and Biomin C glasses.

In the case of the glass containing fluoride ions (e.g., Biomin F), fluoride is removed from the glass and absorbed by apatite, and the most stable crystals of fluorapatite are formed^17,18^. In the present study, the release of fluorine ions during the first 24 h at pH 4 was 7.05 ± 1.06 mg/L.

The role of fluorine ions in methacrylates is important, which, after being released from glass, take part in the formation of fluorapatite on the surface of the teeth or on the border of the teeth in the composite material^26^. Biomin F contains CaF_2_ as the raw material and may be a source of fluorine ions with cariostatic properties^17^. In the present study, F ions from Biomin F were quickly washed out within the first 24 h from the saliva solution.

The rate and quantity of ions released from glass particles are mainly influenced by their size, which, as shown in the present study, should be around 5 μm. Hence, glass particles with the selected diameter were in this study. Too large molecules react slowly, whereas small molecules have a very large surface area. The ion release process is also influenced by the degree of crosslinking of the methacrylate resins. In composite materials, 2–3 functionals are used as the organic phase. Methyl methacrylate has one bond capable of radical polymerization^20,32,33^. Therefore, it is less crosslinked than composite materials.

In the present study, such materials were created by mixing PMMA (Superacryl Plus powder—PMMA) with four different types of glasses, which underwent gradual hydrolysis under the influence of water, releasing ions into the environment.

## Conclusions

- The acrylic material synthesized in this study, after modification with bioactive glass, met the ISO 20795-1: 2013 standard in terms of flexural strength as well as sorption.
- The acrylic resin prepared with 10% addition of various active glasses can release calcium and silicon phosphor ions.
- In the case of Biomin F glass, the release of fluorine ions in an acidic environment was very dynamic (it occurred within the first 24 h). In a neutral environment, ions were released gradually over a period of 42 days.
- Acrylic resins modified with 10% Biomin C and S53P4 glasses can be valuable sources of calcium cations and phosphate anions under acid conditions (pH 4) over a period of 42 days.

## Data Availability

The datasets used and/or analyzed in the present study are available from the corresponding author on reasonable request.
